# Surgical Repair of Bile Duct Injuries Due to Cholecystectomy—An Experience from a Referral Center in Slovenia

**DOI:** 10.3390/life15060874

**Published:** 2025-05-29

**Authors:** Irena Plahuta, Špela Turk, Barbara Lovrenčič Petreski, Tomislav Magdalenić, Stojan Potrč, Arpad Ivanecz

**Affiliations:** 1 Clinical Department of Abdominal and General Surgery, University Medical Centre Maribor, Ljubljanska Ulica 5, 2000 Maribor, Slovenia; 2 Department of Surgery, Faculty of Medicine, University of Maribor, Taborska Ulica 8, 2000 Maribor, Slovenia

**Keywords:** cholecystectomy, bile duct injury, biliodigestive anastomosis, patency, morbidity, mortality

## Abstract

Background: Bile duct injury (BDI) during (laparoscopic) cholecystectomy has an incidence of up to 1.5%. This retrospective study aimed to report the outcomes of surgical repair of BDI due to these procedures at a tertiary referral center. Methods: A retrospective review of patients’ records was conducted. The BDI’s clinical presentations, Strasberg classification, surgical repairs, and outcomes were reported. Results: From 2003 to 2024, 47 BDIs were identified. In total, 34.0% were recognized intraoperatively. The BDI types included Strasberg types B (2, 4.3%), C (5, 10.6%), D (11, 23.4%), E1 (4, 8.5%), E2 (12, 25.5%), E3 (5, 10.6%), E4 (3, 6.4%), and E5 (5, 10.6%). The T-tube group included 6 (12.8%) patients, the primary repair and T-tube group included 10 (21.3%) patients, and the Biliodigestive anastomosis group included 31 (65.9%) patients. The overall morbidity rate was 40.4%, the major morbidity rate was 21.3%, and the mortality rate was 4.3%. Grade A patency was achieved in 95.6% of patients. In the Biliodigestive anastomosis group, the actuarial 1-, 5- and 10-year grade A patency rates were 77.0%, 70.0%, and 70.0%, respectively. Conclusion: The rate of BDI remains stable. The outcomes of repairs in terms of complications and patency rates are comparable to those in other reports.

## 1. Introduction

The first cholecystectomy was performed by Carl Johann August Langenbuch in 1882 in Berlin, and the first laparoscopic cholecystectomy was performed in 1985 by Erich Mühe in Böblingen, Germany [1,2]. Since then, cholecystectomy has been one of the most routinely performed procedures in surgery, and the laparoscopic approach is the gold standard for patients with symptomatic cholecystolithiasis [3].

Bile duct injuries (BDIs) still occur during these procedures, with an incidence of up to 1.5% [3]. Risk factors have been divided into three categories: factors dependent on the surgeon (false visual perception, surgical technique, and learning curve), the patient (anatomical variants, inflammatory processes, obesity), and the institution (equipment and availability of fluoroscopy) [4,5].

A critical view of safety (CVS) was introduced in 1995 to improve the safety of laparoscopic cholecystectomies [6,7]. It failed only 1 in 300 times, which means it worked 299 out of 300 times [7,8]. About 20% of surgeons accepted the core CVS principles but struggled to meet all three criteria [6]. In addition to identifying the hepatocystic triangle and creating two “windows” in it, it is pivotal to mobilize the lower part of the gallbladder from the cystic plate [3]. The surgeon can confirm that the dissected structures enter the gallbladder wall and not the liver parenchyma [3,6]. In addition to the operative notes, it is important to use a high-quality imaging method to document the CVS [3,6]. Furthermore, Rouviere’s sulcus (or *incisura hepatis dextra* or *Gans incisura*) is an anatomical landmark present in at least 80% of people [9]. Identifying Rouviere’s sulcus and preparing structures delineating Calot’s triangle above the level of that sulcus contributes to performing a safer cholecystectomy [10,11].

BDIs can endanger patients or significantly impair their quality of life; therefore, selecting the most effective treatment option for BDI is crucial [3,12,13]. BDIs are often unnoticed during index surgery [14]. When the injury is detected intraoperatively, immediately reconstructing bile ducts is sometimes possible [15]. When injury is suspected in the postoperative period, the type of injury must be identified using magnetic resonance cholangiopancreatography (MRCP), percutaneous transhepatic cholangiography, or endoscopic retrograde cholangiopancreatography (ERCP) [14]. In patients undergoing bile duct reconstruction due to BDI, preoperative or intraoperative Doppler ultrasonography can help to determine if arterial reconstruction is needed. Vascular continuity can also be assessed using magnetic resonance or computed tomography angiography [16].

Simple postoperative leaks can be managed through either drainage alone or combining drainage with the ERCP placement of biliary stents [14]. Some advanced centers, equipped with skilled gastroenterologists, treat selected patients with complex injuries through percutaneous–endoscopic rendezvous procedures, showing promising long-term outcomes [14]. Biliodigestive anastomosis (BDA) is the gold standard for treating severe and complete transection of bile ducts [17,18,19].

This retrospective study aimed to report the outcomes of the surgical repair of BDI due to (laparoscopic) cholecystectomy at a tertiary referral center.

## 2. Materials and Methods

A retrospective review of a database of patients who underwent treatment for BDI at our tertiary referral center from January 2003 to December 2024 was performed. Our department is one of two tertiary referral centers in Slovenia that surgically repair major BDI. We perform about 300 elective and emergency cholecystectomies yearly. Additionally, our dedicated hepato-pancreato-biliary team consists of five surgeons performing about 130 resections annually, including 30 pancreaticoduodenectomies [20,21].

All patients who underwent surgical repair for BDI due to the cholecystectomy were included in the research. Patients who endured BDI during ERCP and surgeries other than cholecystectomy (bile duct resections for cysts, neoplasms, and choledocholithiasis; liver resections for liver tumors and abscess drainages; surgery for gallbladder or perihilar cholangiocarcinoma; and pancreaticoduodenectomies) were excluded. Patients whose BDIs were repaired via ERCP were also excluded, as suggested by Cho et al. [22].

The patients’ demographics, preoperative clinical characteristics, intraoperative details, and postoperative outcomes were retrieved from the database.

Surgical repair was always performed through an open approach. The following repairs were performed:

- T-tube placement.
- Primary sutures of the bile ducts with a T-tube placement.
- BDA was performed in a Roux-en-Y manner for complete transections of the bile ducts. The surgical technique was applied as described by the reference [23].

BDI was graded according to the Strassberg classification, as proposed by the World Society of Emergency Surgery [3,24]. Cholangitis was defined according to the Tokyo Guidelines 2018 [25].

The time frame of the repair was reported during the index surgery, as well as in the early (days 1–7), intermediate (1–6 weeks), and late (>6 weeks) periods post-surgery [15].

Postoperative morbidity was graded according to the Clavien–Dindo classification [26]. The most severe complication was taken into account [26]. Grades 1 and 2 indicate minor complications that require medical therapies for treatment. Severe complications encompassed grades ≥ 3a. Grade 3a means that interventional radiological or endoscopic procedures without general anesthesia are needed. Grade 3b necessitates the aforementioned and surgical procedures under general anesthesia. Grades 4a and 4b involve organ support, and grade 5 signifies mortality [26].

The primary endpoint was primary patency after BDI repair. “Patency” is defined as an open, functional biliary tree on the 90th day after surgical repair [22]. An open functional biliary tree means that it lacks stents and the need for invasive interventions. This definition applies to a patient who, after completing treatment, has no episodes of jaundice, cholangitis, external biliary fistula, or liver abscesses [22]. The grading of patency, its actual rates, and its actuarial duration were calculated as proposed by Cho et al. [22,27].

The secondary endpoints were postoperative outcomes (overall morbidity, severe morbidity, mortality).

IBM SPSS for Windows Version 29.0.0.0 (IBM Corporation, Armonk, NY, USA) was used for conducting statistical computations. Percentages were reported to one decimal place.

Categorical variables were displayed as numbers (percentages). Continuous variables were expressed as medians (interquartile range) [28].

Patients underwent check-ups according to their clinical state. This study was concluded on 31 December 2024 or upon the patient’s death. The median follow-up period was calculated using the reverse Kaplan–Meier method and expressed as months (confidence interval). The Kaplan–Meier curves show the primary patency intervals, and the Survival Tables were used to calculate the 1-, 3-, 5-, and 10-year actuarial patencies [22,29].

The Institutional Ethics Committee approved this study (UKC-MB-KME-42/24). Before undergoing surgery, patients consented to their anonymous data being used for research. Their records were anonymized and deidentified before analysis. Informed consent for publication was obtained from all identifiable human participants. All methods were performed following the relevant guidelines and regulations.

## 3. Results

**Figure 1:**
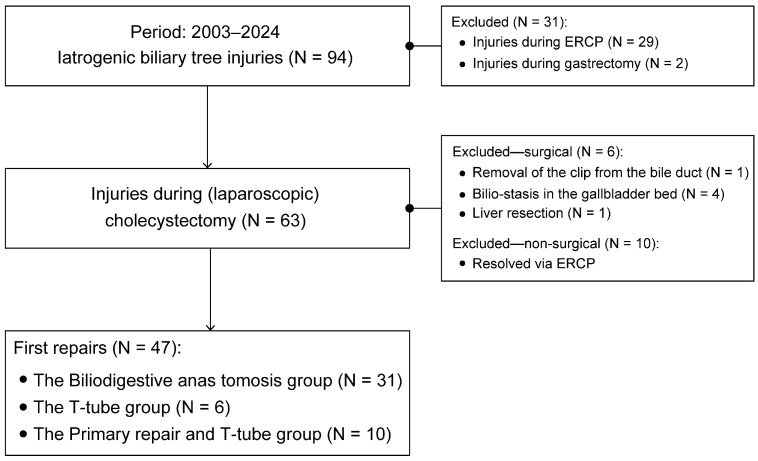
A study flow chart. The search excluded bile duct resections for cysts, neoplasms, and choledocholithiasis; liver resections for liver tumors and abscess drainages; surgery for gallbladder or perihilar cholangiocarcinoma; and pancreaticoduodenectomies. ERCP: Endoscopic retrograde cholangiopancreatography.

Patient data were retrieved from various sources, and there were inconsistent reporting and coding of diseases, complications, and procedures. Therefore, the search was conducted following the method shown in Figure 1.

From 2003 to 2024, 94 patients with BDIs were treated at our department. They were either operated on by us or referred to us by various gastroenterology departments and four secondary-level hospitals.

A total of 63 patients with BDIs due to cholecystectomy were identified. Six patients whose surgical treatment could not be assigned to any of the groups were excluded from further analysis (Figure 1). In addition, ten patients whose BDIs were resolved via ERCP were excluded on the basis of their injuries being resolved non-surgically [22].

A total of 47 patients with BDIs caused during cholecystectomy were further analyzed. Of these, 25 injuries (53.2%) occurred within our department, while other hospitals referred 22 patients (46.8%) to us, and 16 (72.2%) of them needed BDA.

The incidence of BDI in our department was tracked from 2012 to 2024. During this period, we performed 3724 open or laparoscopic cholecystectomies, which resulted in 29 cases of BDI, 10 of which required surgical reconstruction (6 BDAs and 4 primary repairs). That gave an overall incidence of 0.8% and an incidence of major injuries of 0.3%.

These 47 patients were categorized into three groups based on the type of primary repair for BDI. The T-tube group included 6 (12.8%) patients, the primary repair and T-tube group included 10 (21.3%) patients, and the Biliodigestive anastomosis group included 31 (65.9%) patients. Their basic characteristics are detailed in Table 1.

The indications for surgery were acute cholecystitis in 14 (29.8%) cases, persistent biliary colic in 3 (6.4%) cases, symptomatic cholecystolithiasis in 12 (25.5%) cases, and chronic cholecystitis in 18 (38.3%) cases.

Operative reports were available in only 25 (53.2%) cholecystectomy cases. Among them, Calot’s triangle was dissected first in 16 cases, the cystic duct was identified as such in 21 cases, and the cystic artery was identified as such in 11 cases. A CVS was mentioned in 14 cases.

Table 2 shows the clinical presentations of BDI and the time frame of the repair. In total, 34.0% of BDIs were identified during cholecystectomy. Jaundice was the second-most common symptom, occurring in a quarter of cases, followed by bile in drain (23.4%) and biliary peritonitis (14.9%). BDI was recognized due to a concomitant lesion of the right hepatic artery in one (2.1%) patient. Three patients in the late repair period were operated on several months (9, 26, and 173) after cholecystectomy due to stenosis of the common bile duct.

Injuries were graded according to the Strassberg classification (Table 3) [24]. Concomitant vascular injury was present in nine (19.1%) cases, a cystic artery was injured in one case (11.1%), and a right hepatic artery was injured in eight (88.9%) cases.

Repairs were BDA in 31 cases, T-tube placement in 6 cases, and primary repair of a bile duct and a T-tube placement in 10 cases. The cystic artery was properly ligated, and the right hepatic artery was reconstructed in all cases. The complications of the repairs according to the Clavien–Dindo classification are given in Table 4.

The description of severe morbidity is as follows: Clavien–Dindo grade 3a complications were percutaneous drainages of intra-abdominal fluid collections. One patient was reoperated on because of laparotomy dehiscence and another for the surgical lavage of biliary peritonitis (grade 3b). One patient was admitted to the intensive care unit due to respiratory insufficiency (grade 4a). One patient was admitted due to hemorrhagic shock, and two patients were admitted due to septic shock (grade 4b). One patient from the BDA group died of multiple organ dysfunction syndrome, and one patient from the primary repair and T-tube group died of pneumonia.

Table 5 shows severe morbidity (Clavien–Dindo ≥ 3a) according to the time frame of the repair.

When short-term outcomes were compared according to whether the cholecystectomy was elective or emergency, the Clavien–Dindo ≥ 3a grades after repairs were 16.7% and 29.4%, respectively. Two patients who died came from both groups.

**Figure 2:**
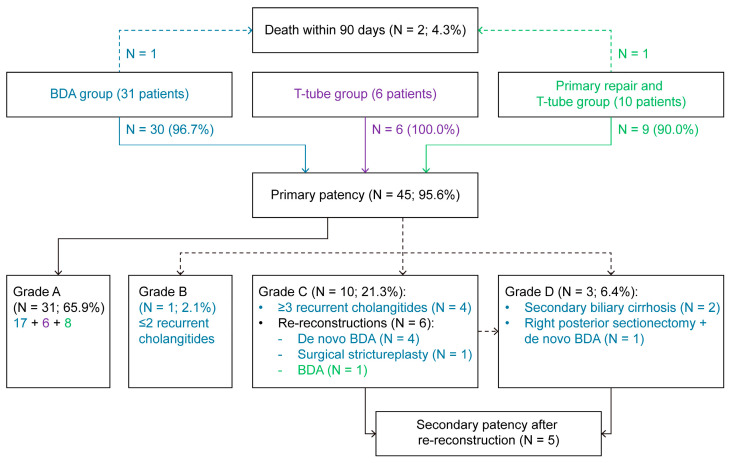
Patency of repairs of bile duct injuries. BDA: Biliodigestive anastomosis. Blue: BDA group; Purple: T-tube group; Green: Primary repair and T-tube group.

The patency results after the BDI repairs are shown in Figure 2 [22].

Two patients never reached primary patency because they died within 90 days after the surgical repair of BDI. Four patients with recurrent cholangitis were frequently treated with antibiotics.

One patient had a surgical strictureplasty and six patients underwent re-reconstructions of bile ducts. In the patient from the primary repair and T-tube group, BDA was created after ten endoscopic retrograde cholangiopancreatography procedures. In four cases, BDA was resected and created de novo. One patient underwent a concomitant posterior sectionectomy due to limited secondary liver cirrhosis. Of these seven patients, five achieved secondary patency and had no issues. Two patients developed secondary liver cirrhosis. The median gamma-glutamyl transferase and alkaline phosphatase values of patients with secondary biliary cirrhosis were 3.17 ukat/L and 2.52 ukat/L, respectively. One patient died of heart failure, and one patient remained on the waiting list for liver transplantation.

**Figure 3:**
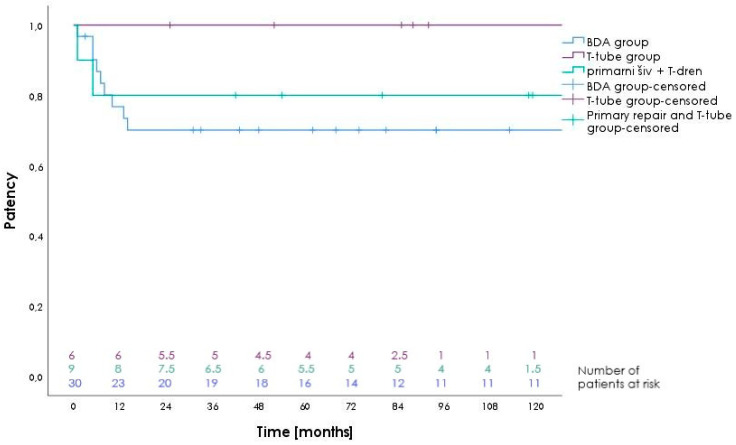
Kaplan–Meier curves of actuarial primary patency dependent on time. BDA: Biliodigestive anastomosis.

The median follow-up period was 94 (confidence interval 57.6–130.5) months. Figure 3 shows a Kaplan–Meier graph of actuarial primary patency dependent on time.

Table 6 shows the actuarial primary patency rates 1, 3, 5, and 10 years after repairs.

Five patients achieved secondary patency after re-reconstruction; its median duration was 106 months.

## 4. Discussion

This study analyzed 47 patients who underwent surgical repair of BDI due to cholecystectomy. The 90-day overall morbidity rate was 40.4%, the major morbidity rate was 21.3%, and the mortality rate was 4.3%. Grade A patency was achieved in 95.6% of patients. A total of 65.9% of patients remained in that grade, while 2.1% of patients slid into grade B, 21.3% of patients were reassigned to grade C, and 6.4% were reassigned to grade D. In the BDA group, the actuarial 1-, 5- and 10-year grade A patency rates were 77.0%, 70.0%, and 70.0%, respectively.

The majority of BDIs are iatrogenic, and they occur during abdominal surgeries or interventions such as endoscopic or percutaneous cannulation of the biliary tree [30,31]. In the last 21 years, our department has treated at least 94 iatrogenic BDIs, and 63 of them were due to cholecystectomy (Figure 1). A similar case series showed that most BDI referrals to a tertiary center were usually very serious complications [32,33]. In this study, 72.2% of referred patients needed BDA. Patients with BDI must be referred immediately to a surgeon experienced in managing BDI at a facility with a hepatobiliary multispecialty team. If a timely referral is not feasible, consultation with a surgeon experienced in managing BDI should be carried out [3,12].

The incidence of BDI was low (0.1–0.2%) during the era of open cholecystectomy, but this rate has tripled (0.4–0.6%) following the global implementation of the laparoscopic approach [34]. The incidence of major BDI in our department between 2012 and 2024 was 0.3%, and the overall incidence was 0.8%. That aligns with nationwide prospective databases that indicate figures of up to 1.5% [3,6,33,34,35].

Evaluating the risk factors for BDIs within our cohort presents certain challenges, particularly given the diversity of the five hospitals and various surgical teams involved. Among the patients, more than half were female, with a median age of 65 and a median body mass index of 29 kg/m². Chronic cholecystitis was the most common indication for surgery, accounting for 38.3% of cases. Additionally, two-thirds of the cholecystectomies were performed on an elective basis. Approximately 85.0% of these procedures were started laparoscopically (Table 1).

The most common etiological factor for BDI is misidentifying the common bile duct as the cystic duct; aberrant anatomy and difficult pathology are less commonly responsible for BDI [34]. This was also true in our case series, where the common bile duct was identified as the cystic duct in 29 cases (61.7%) (Strasberg type E injuries). In six (12.8%) cases, the right posterior sectoral duct was identified as the cystic duct (Strasberg type B and C injuries). A bile duct variation in the right posterior sector duct connecting directly to the cystic duct was present only in one patient (2.1%). However, out of our study’s 25 available operative reports, CVS was only mentioned in 14 cases, although implementing that rule significantly decreases the rate of BDI [3].

Performing a cholecystectomy with two surgeons present is often not feasible in many settings. However, having a second surgeon can be beneficial, especially when the dissection is stalled, the anatomy is unclear, or the operating conditions are deemed “difficult”. Consulting a more experienced surgeon can provide a critical moment in preventing BDI or its early recognition [3,12].

Intraoperative cholangiography (IOC) is an imaging technique used during cholecystectomy to identify choledocholithiasis and clarify the anatomy of the bile ducts [3]. However, routine use of IOC is not recommended, as it does not significantly reduce the risk of BDI [3,35]. Additionally, BDI can still occur after IOC due to misinterpreting the cholangiogram [3]. IOC is advisable in certain situations, particularly when the anatomy of the bile ducts is unclear. It may also be beneficial for patients with acute cholecystitis or a history of it, even though it may prolong the duration of surgery [3,35]. If there is concern about a potential BDI, performing IOC can assist in diagnosing and treating it on time [3]. Another technique is indocyanine green fluorescence cholangiography [36]. This method involves injecting a dye to visualize the bile duct structures without using X-rays [36]. Studies suggest that this technique may help to prevent BDI and is effective for acute and chronic gallbladder issues, especially when IOC cannot be performed [3,36].

Table 2 shows the clinical presentation and time frame of the repair of BDI. One-third of the injuries were recognized during cholecystectomy. Rates of intraoperatively recognized BDI ranged from 16% to 46% [34,37]. There was a low rate of biliary peritonitis in the BDA group (6.5%) and a high rate (40.0%) in the primary repair and T-tube group. In that group, biliary peritonitis was present in 40% of cases, and the repairs were completed after 7 days. When BDI is suspected in the postoperative period, a computed tomography scan of the abdomen should be conducted, and in cases of significant peritoneal fluid accumulation, percutaneous drainage should be carried out [14]. Then, a work-up with MRCP or ERCP must be carried out to delineate the anatomy to plan the repair [14].

The ERCP with stenting is a viable approach for treating BDI, but we excluded patients who underwent this repair, as proposed by Cho et al. [22]. Namely, patients who can only receive surgical treatment should be reported in the surgical case series. Those eligible only for non-surgical treatment can be included in case series that study endoscopic or interventional radiology methods [22]. However, mixing these patients in studies that compare surgical and non-surgical techniques is not appropriate [22]. That does not mean that we overlooked the fact that some complex injuries can only be treated with surgery and that there are patients who, while not suitable for surgery, can still be compatible with non-surgical options [22]. However, including these patients in comparative studies would make the results difficult to interpret [15].

The delayed recognition of BDI, injuries of the right hepatic artery (eight cases, or 88.9% of concomitant vascular injuries), and complications in repair contributed to the overall severe morbidity (Clavien–Dindo grade ≥ 3a) of 21.3% and mortality of 4.3% (Table 4) [15]. Reported rates of severe morbidity were as high as 43.0%, and mortality was as high as 4.6% [3,19,27,38].

The treatment of these injuries after the index operation depends on the severity of the injury, the time of diagnosis, and the patient’s general condition. Late detection of the injury is associated with reduced survival [39]. In our study, the severe morbidity rate was 6.25% when the repair was performed during index surgery and increased upon early (33.3%) and intermediate repair (30%) (Table 5). Especially in the BDA group, the rates of complications skyrocketed from 11.1% to 30.0% and up to 50% in the intermediate time frame of the repair. On the contrary, the multicenter study showed that the timing of biliary reconstruction with hepaticojejunostomy does not have any impact on severe postoperative complications [15].

Surgical repairs in our institution were always carried out via an open approach. Currently, it is too early to recommend using laparoscopic and robotic tools for BDA in BDI repair [13]. However, the perspectives of minimally invasive repairs are quite promising for patients’ quality of life [13].

Classification systems based on the biliary injury location include the first one published by Bismuth in 1982, that of Strasberg published in 1995, and others [3,24]. In 2013, the European Association for Endoscopic Surgery proposed the ATOM (Anatomic, Time Of detection, Mechanism) classification [3,40]. It covers all possible injuries by combining bile tract anatomical damage, vascular injury, the timing of detection, and damage mechanism. The intention was to standardize BDI definitions and facilitate data collection to develop preventive measures [40]. The main disadvantage of ATOM is that it may be too intricate and time-consuming for routine clinical practice [3]. We reported BDI according to the Strasberg classification (Table 3), as proposed by the World Society of Emergency Surgery [3]. Furthermore, we found this classification more useful than ATOM because we often classified BDI retrospectively according to the repair performed.

Figure 2 shows the results of actual patency. As expected, minor injuries repaired by a T-tube placement had an excellent outcome [3]. The results from the primary repair and T-tube group were less favorable, where one patient needed BDA. In the BDA group, 96.7% of patients attained primary patency, but only 56.6% remained in that grade.

Actuarial primary patency rates in the BDA group were 77.0%, 70.0%, and 70.0% at 1, 5, and 10 years, respectively (Figure 3 and Table 6). In the study by Cho et al., primary patency was achieved at 94.0%, and the 5- and 10-year rates were 92% [22]. Martínez–Mier reported a primary patency of 93.4%. The 1-, 5-, and 10-year rates were 91.2%, 53.9%, and 53.9%, respectively [41]. Lindemann et al. found a primary patency at 98.1%. The 1-, 5-, and 10-year retention rates were 96.4%, 90.6%, and 81.5% [42]. Otto et al. reported that actuarial grade A patency rates at 5 and 10 years were 88% and 74%, respectively [27].

The limitation of this study was the notably low number of patients, which precluded the use of advanced multivariate models and calculations. Another limitation was the retrospective nature of the study, with confined access to the patients’ documentation. A useful future research direction would be to establish a nationwide registry of cholecystectomies to study their feasibility and safety in our country [12].

To conclude, the rate of BDI remains stable, and our center encourages colleagues to refer patients with confirmed or suspected BDI to a tertiary center. The outcomes in terms of complications and patency rates are comparable to those in other reports.

## Figures and Tables

**Table 1:** Basic characteristics of patients who endured bile duct injury during cholecystectomy.

Variable	Total (N = 47)	BDA Group (N = 31)	T-Tube Group (N = 6)	Primary Repair and T-Tube Group (N = 10)
Male sex	23 (48.9)	17 (54.8)	3 (50.0)	3 (30.0)
Age [years]	65 (18)	60 (20)	69 (16)	68 (13)
BMI [kg/m^2^]	29 (7)	29 (5)	36 (3)	28 (2)
ASA 3 and 4	14 (29.8)	10 (32.3)	2 (33.3)	2 (20.0)
Method				
open	7 (15.0)	4 (12.9)	1 (16.7)	2 (20.0)
laparoscopic	20 (42.5)	13 (41.9)	3 (50.0)	4 (40.0)
conversion	20 (42.5)	14 (45.2)	2 (33.3)	4 (40.0)
Elective surgery	30 (63.8)	19 (61.3)	4 (66.7)	7 (70.0)

ASA: American Society of Anesthesiologists Score; BDA: Biliodigestive anastomosis; BMI: body mass index.

**Table 2:** The clinical presentations of bile duct injuries and the time frame of the repair in 47 patients.

Clinical Presentation	Total (N = 47)	BDA Group (N = 31)	T-Tube Group (N = 6)	Primary Repair and T-Tube Group (N = 10)
During index surgery	16 (34.0)	9 (29.0)	2 (33.3)	5 (50.0)
Icterus/holangitis	12 (25.5)	12 (38.7)	0 (0.0)	0 (0.0)
Bile in drain	11 (23.4)	7 (22.6)	3 (50.0)	1 (10.0)
Biliary peritonitis	7 (14.9)	2 (6.5)	1 (16.7)	4 (40.0)
Hemorrhagic shock	1 (2.1)	1 (3.2)	0 (0.0)	0 (0.0)
**Time of the repair**				
During index surgery	16 (34.0)	9 (29.0)	2 (33.3)	5 (50.0)
Early (day 1–7)	15 (31.9)	10 (61.3)	4 (66.7)	1 (10.0)
Intermediate (1–6 weeks)	10 (21.3)	6 (19.4)		4 (40.0)
Late (>6 weeks)	6 (12.7)	6 (19.4)		

BDA: Biliodigestive anastomosis.

**Table 3:** Grading of injuries according to the Strassberg classification.

Grade of Injury	Total (N = 47)	BDA Group (N = 31)	T-Tube Group (N = 6)	Primary Repair and T-Tube Group (N = 10)
B	2 (4.3)	2 (6.5)		
C	5 (10.6)			5 (50.0)
D	11 (23.4)		6 (100.0)	5 (50.0)
E1	4 (8.5)	4 (12.9)		
E2	12 (25.5)	12 (38.7)		
E3	5 (10.6)	5 (16.1)		
E4	3 (6.4)	3 (9.7)		
E5	5 (10.6)	5 (16.1)		

B: occluded right posterior sectoral duct; C: bile leak from divided right posterior sectoral duct; D: bile leak from main bile duct without major tissue loss; E1 and E2: transected main bile duct with a stricture more than 2 cm from the hilus (E1) or less than 2 cm from the hilus (E2); E3 and E4: stricture of the hilus with right and left duct in communication (E3) or without communication (E4); E5: stricture of the main bile duct and the right posterior sectoral duct [24]. BDA: Biliodigestive anastomosis.

**Table 4:** The grading of complications according to the Clavien–Dindo classification.

Grade of Injury	Total (N = 47)	BDA Group (N = 31)	T-Tube Group (N = 6)	Primary Repair and T-Tube Group (N = 10)
CD 1	2 (4.3)	2 (6.5)		
CD 2	7 (14.9)	5 (16.1)		2 (20.0)
CD 3a	2 (4.3)	1 (3.2)	1 (16.7)	
CD 3b	2 (4.3)	2 (6.5)		
CD 4a	1 (2.1)	1 (3.2)		
CD 4b	3 (6.4)	2 (6.5)		1 (10.0)
CD 5	2 (4.3)	1 (3.2)		1 (10.0)
Overall morbidity	19 (40.4)	14 (45.2)	1 (16.7)	4 (40.0)
Severe morbidity ≥ CD 3a	10 (21.3)	7 (22.6)	1 (16.7)	2 (20.0)

BDA: Biliodigestive anastomosis; CD: Clavien–Dindo classification.

**Table 5:** The rates and grading of severe morbidity (Clavien–Dindo ≥ 3a) according to the time frame of the repair.

Time Frame	Total (N = 47)	BDA Group (N = 31)	T-Tube Group (N = 6)	Primary Repair and T-Tube Group (N = 10)
During index surgery	1 (6.25%)	11.1% 3b		
Early (day 1–7)	5 (33.3%)	30.0% 3b, 2 pts 4b	25.0% 3a	20.0% 5
Intermediate (1–6 weeks)	3 (30.0%)	50.0% 3a, 4a, 5		25.0% 4b
Late (>6 weeks)				

BDA: Biliodigestive anastomosis.

**Table 6:** The actuarial primary patency rates 1, 3, 5, and 10 years after repairs.

	BDA Group (N = 31)	T-Tube Group (N = 6)	Primary Repair and T-Tube Group (N = 10)
1 year	77.0%	100.0%	80.0%
3 years	70.0%	100.0%	80.0%
5 years	70.0%	100.0%	80.0%
10 years	70.0%	100.0%	80.0%

BDA: Biliodigestive anastomosis.

## Data Availability

Due to the patients’ privacy, the data presented in this study are available upon request from the corresponding author.

## References

[1] Sparkman R.S. (1982). 100th Anniversary of the First Cholecystectomy: A Reprinting of the 50th Anniversary Article From the Archives of Surgery, July 1932. Arch. Surg..

[2] Blum C.A., Adams D.B. (2011). Who did the first laparoscopic cholecystectomy?. J. Minim. Access Surg..

[3] de’Angelis N., Catena F., Memeo R., Coccolini F., Martínez-Pérez A., Romeo O.M., De Simone B., Di Saverio S., Brustia R., Rhaiem R. (2021). 2020 WSES guidelines for the detection and management of bile duct injury during cholecystectomy. World J. Emerg. Surg..

[4] Way L.W., Stewart L., Gantert W., Liu K., Lee C.M., Whang K., Hunter J.G. (2003). Causes and prevention of laparoscopic bile duct injuries: Analysis of 252 cases from a human factors and cognitive psychology perspective. Ann. Surg..

[5] Pekolj J., Pekolj J., Ardiles V., Glinka J. (2022). Introduction. Fundamentals of Bile Duct Injuries: From Prevention to Multidisciplinary Management.

[6] Manatakis D.K., Antonopoulou M.I., Tasis N., Agalianos C., Tsouknidas I., Korkolis D.P., Dervenis C. (2023). Critical View of Safety in Laparoscopic Cholecystectomy: A Systematic Review of Current Evidence and Future Perspectives. World J. Surg..

[7] Strasberg S.M. (2017). A perspective on the critical view of safety in laparoscopic cholecystectomy. Ann. Laparosc. Endosc. Surg..

[8] Daly S.C., Deziel D.J., Li X., Thaqi M., Millikan K.W., Myers J.A., Bonomo S., Luu M.B. (2016). Current practices in biliary surgery: Do we practice what we teach?. Surg. Endosc..

[9] Dahmane R., Morjane A., Starc A. (2013). Anatomy and surgical relevance of Rouviere’s sulcus. Sci. World J..

[10] Lockhart S., Singh-Ranger G. (2018). Rouviere’s sulcus-Aspects of incorporating this valuable sign for laparoscopic cholecystectomy. Asian J. Surg..

[11] Basukala S., Thapa N., Tamang A., Shah K.B., Rayamajhi B.B., Ayer D., Karki S., Basukala B., Sharma S., Dhakal S. (2022). Rouviere’s sulcus—An anatomical landmark for safe laparoscopic cholecystectomy: A cross-sectional study. Ann. Med. Surg..

[12] Brunt L.M., Deziel D.J., Telem D.A., Strasberg S.M., Aggarwal R., Asbun H., Bonjer J., McDonald M., Alseidi A., Ujiki M. (2020). Safe Cholecystectomy Multi-society Practice Guideline and State of the Art Consensus Conference on Prevention of Bile Duct Injury During Cholecystectomy. Ann. Surg..

[13] Marichez A., Adam J.P., Laurent C., Chiche L. (2023). Hepaticojejunostomy for bile duct injury: State of the art. Langenbecks Arch. Surg..

[14] Bonds M., Rocha F., Jarnagin W.R., Allen P.J., Chapman W.C., D’Angelica M.I., DeMatteo R.P., Do R.K.G., Vauthey J.-N., Blumgart L.H. (2023). Cholecystectomy techniques and postoperative problems. Blumgart’s Surgery of the Liver, Biliary Tract, and Pancreas.

[15] (2019). Post cholecystectomy bile duct injury: Early, intermediate or late repair with hepaticojejunostomy—An E-AHPBA multi-center study. HPB.

[16] Atay A., Gungor F., Sur Y., Gunes O., Dilek F.H., Karasu Ş., Dilek O.N. (2022). Management of hepatic artery trauma during hepato-pancreato-biliary procedures: Evolving approaches, clinical outcomes, and literature review. Ulus. Travma Acil Cerrahi Derg..

[17] Perera M.T., Silva M.A., Hegab B., Muralidharan V., Bramhall S.R., Mayer A.D., Buckels J.A., Mirza D.F. (2011). Specialist early and immediate repair of post-laparoscopic cholecystectomy bile duct injuries is associated with an improved long-term outcome. Ann. Surg..

[18] de Reuver P.R., Grossmann I., Busch O.R., Obertop H., van Gulik T.M., Gouma D.J. (2007). Referral pattern and timing of repair are risk factors for complications after reconstructive surgery for bile duct injury. Ann. Surg..

[19] Stilling N.M., Fristrup C., Wettergren A., Ugianskis A., Nygaard J., Holte K., Bardram L., Sall M., Mortensen M.B. (2015). Long-term outcome after early repair of iatrogenic bile duct injury. A national Danish multicentre study. HPB.

[20] Plahuta I., Šarenac Ž., Golob M., Turk Š., Ilijevec B., Magdalenić T., Potrč S., Ivanecz A. (2025). Laparoscopic and Open Distal Pancreatectomy-An Initial Single-Institution Experience with a Propensity Score Matching Analysis. Life.

[21] Ivanecz A., Plahuta I., Mencinger M., Perus I., Magdalenic T., Turk S., Potrc S. (2021). The learning curve of laparoscopic liver resection utilising a difficulty score. Radiol. Oncol..

[22] Cho J.Y., Baron T.H., Carr-Locke D.L., Chapman W.C., Costamagna G., de Santibanes E., Dominguez Rosado I., Garden O.J., Gouma D., Lillemoe K.D. (2018). Proposed standards for reporting outcomes of treating biliary injuries. HPB.

[23] Bredbeck B.C., Cho C.S., Jarnagin W.R., Allen P.J., Chapman W.C., D’Angelica M.I., DeMatteo R.P., Do R.K.G., Vauthey J.-N., Blumgart L.H. (2023). Bile duct exploration and biliary-enteric anastomosis. Blumgart’s Surgery of the Liver, Biliary Tract, and Pancreas.

[24] Strasberg S.M., Hertl M., Soper N.J. (1995). An analysis of the problem of biliary injury during laparoscopic cholecystectomy. J. Am. Coll. Surg..

[25] Miura F., Okamoto K., Takada T., Strasberg S.M., Asbun H.J., Pitt H.A., Gomi H., Solomkin J.S., Schlossberg D., Han H.S. (2018). Tokyo Guidelines 2018: Initial management of acute biliary infection and flowchart for acute cholangitis. J. Hepatobiliary Pancreat. Sci..

[26] Clavien P.A., Barkun J., de Oliveira M.L., Vauthey J.N., Dindo D., Schulick R.D., de Santibanes E., Pekolj J., Slankamenac K., Bassi C. (2009). The Clavien-Dindo classification of surgical complications: Five-year experience. Ann. Surg..

[27] Otto W., Sierdziński J., Smaga J., Kornasiewicz O., Dudek K., Zieniewicz K. (2022). Actuarial Patency Rates of Hepatico-Jejunal Anastomosis after Repair of Bile Duct Injury at a Reference Center. J. Clin. Med..

[28] Habibzadeh F. (2017). Statistical Data Editing in Scientific Articles. J. Korean Med. Sci..

[29] Bewick V., Cheek L., Ball J. (2004). Statistics review 12: Survival analysis. Crit. Care.

[30] Molinari M., Garbuzenko D.V. (2016). Traumatic Bile Duct Injuries. Actual Problems of Emergency Abdominal Surgery.

[31] Breznik S., Slanič A., Ivanecz A., Lučev J. (2024). Bilhemia After Percutaneous Liver Tumor Core Biopsy With the Percutaneous Embolization of Bilio-venous Fistula With Coils and Onyx. Cureus.

[32] Bektas H., Schrem H., Winny M., Klempnauer J. (2007). Surgical treatment and outcome of iatrogenic bile duct lesions after cholecystectomy and the impact of different clinical classification systems. Br. J. Surg..

[33] Šileikis A., Žulpaitė R., Šileikytė A., Lukšta M. (2019). Postcholecystectomy bile duct injuries: Evolution of surgical treatment. Pol. Prz. Chir..

[34] Kapoor V.K. (2020). Post-Cholecystectomy Bile Duct Injury.

[35] Törnqvist B., Strömberg C., Akre O., Enochsson L., Nilsson M. (2015). Selective intraoperative cholangiography and risk of bile duct injury during cholecystectomy. Br. J. Surg..

[36] Goldstein S.D., Lautz T.B. (2020). Fluorescent Cholangiography During Laparoscopic Cholecystectomy: Shedding New Light on Biliary Anatomy. JAMA Surg..

[37] Durowicz S., Kozicki I., Ciesielski A., Tarnowski W. (2020). Excision of a part of the bile duct as an iatrogenic injury typical for laparoscopic cholecystectomy—Characteristics, treatment and long-term results, based on own material. Wideochir. Inne Tech. Maloinwazyjne.

[38] Barbier L., Souche R., Slim K., Ah-Soune P. (2014). Long-term consequences of bile duct injury after cholecystectomy. J. Visc. Surg..

[39] Törnqvist B., Strömberg C., Persson G., Nilsson M. (2012). Effect of intended intraoperative cholangiography and early detection of bile duct injury on survival after cholecystectomy: Population based cohort study. BMJ.

[40] Fingerhut A., Dziri C., Garden O.J., Gouma D., Millat B., Neugebauer E., Paganini A., Targarona E. (2013). ATOM, the all-inclusive, nominal EAES classification of bile duct injuries during cholecystectomy. Surg. Endosc..

[41] Martínez-Mier G., Moreno-Ley P.I., Mendez-Rico D. (2020). Factors associated with patency loss and actuarial patency rate following post-cholecystectomy bile duct injury repair: Long-term follow-up. Langenbecks Arch. Surg..

[42] Lindemann J., Krige J.E.J., Kotze U., Jonas E. (2020). Factors leading to loss of patency after biliary reconstruction of major laparoscopic cholecystectomy bile duct injuries: An observational study with long-term outcomes. HPB.

